# Anti-*Cryptosporidium parvum* activity of *Artemisia judaica* L. and its fractions: *in vitro* and *in vivo* assays

**DOI:** 10.3389/fmicb.2023.1193810

**Published:** 2023-07-03

**Authors:** Shahira A. Ahmed, Enas E. Eltamany, Mohamed S. Nafie, Sameh S. Elhady, Panagiotis Karanis, Amira B. Mokhtar

**Affiliations:** ^1^Department of Medical Parasitology, Faculty of Medicine, Suez Canal University, Ismailia, Egypt; ^2^Department of Pharmacognosy, Faculty of Pharmacy, Suez Canal University, Ismailia, Egypt; ^3^Department of Chemistry (Biochemistry Program), Faculty of Science, Suez Canal University, Ismailia, Egypt; ^4^Department of Natural Products, Faculty of Pharmacy, King Abdulaziz University, Jeddah, Saudi Arabia; ^5^Center for Artificial Intelligence in Precision Medicines, King Abdulaziz University, Jeddah, Saudi Arabia; ^6^University of Cologne, Medical Faculty and University Hospital, Cologne, Germany; ^7^Department of Basic and Clinical SciencesUniversity of Nicosia Medical School, Nicosia, Cyprus

**Keywords:** *Cryptosporidium* sp., *Artemisia judaica* L., drug discovery and public health, MTT assay, comet assay, *in vivo*, *in vitro*

## Abstract

**Background:**

This study investigates the toxic activity of *Artemisia judaica* ethanolic extract (ArEx) as well as its phenolic fraction (ArPh), and terpenoid fraction (ArT) against *Cryptosporidium parvum* (*C. parvum*) oocysts.

**Methods:**

Over a 4 months period, estimation of the total phenolic (TPC), total flavonoids (TFC), and total terpenoids contents (TTC) in ArEx; investigation of the *in vitro* antioxidant activity of ArEx, ArPh, and ArT; evaluation of ArEx, ArPh, and ArT toxic activity against *C. parvum* oocysts using MTT assay; parasitological analysis on ArPh-treated *C. parvum* oocysts and comet assay were performed both *in vitro* and *in vivo* (infectivity).

**Results:**

The ArEx TPC, TFC, and TTC was 52.6 ± 3.1 mgGAE/g, 64.5 ± 3.1 mg QE/g, and 9.5 ± 1.1 mg Linol/g, respectively. Regarding the phytochemical *in vitro* antioxidant activity, the ArPh exhibited the highest antioxidant activity compared to the ArEx and ArT. The ArPh showed promising free radical scavenging activity of DPPH and ABTS^•+^ with IC_50_ values of 47.27 ± 1.86 μg/mL and 66.89 ± 1.94 μg/mL, respectively. Moreover, the FRAP of ArPh was 2.97 ± 0.65 mMol Fe^+2^/g while its TAC was 46.23 ± 3.15 mg GAE/g. The ArPh demonstrated toxic activity against *C. parvum* oocysts with a potent IC_50_ value of 31.6 μg/mL compared to ArT (promising) and ArEx (non-effective). ArPh parasitological analysis demonstrated MIC_90_ at 1000 μg/ml and effective oocysts destruction on count and morphology. ArPh fragmented oocysts nuclear DNA in comet assay. Beginning at 200 μg/mL, ArPh-treated oocysts did not infect mice.

**Conclusion:**

To combat *C. parvum* infection, the phenolic fraction of *A. judaica* L. shows promise as an adjuvant therapy or as a source of potentially useful lead structures for drug discovery.

## 1. Introduction

The waterborne pathogen *Cryptosporidium* sp. is widely recognized as having medical importance for both immunocompetent and immunocompromised patients. Around 905 waterborne and 25 foodborne outbreaks are reported by *Cryptosporidium* sp. worldwide (Baldursson and Karanis, 2011; Efstratiou et al., 2017; Ahmed and Karanis, 2018b).

The most common transmission routes are contaminated food or water consumption, zoonotic transmission, person-to-person contact, and the underestimated but critical route of airborne transmission (Ahmed et al., 2018; Ahmed and Karanis, 2020). At least 47 species (Ježková et al., 2021; Zahedi et al., 2021) and more than 120 genotypes of *Cryptosporidium* sp. have been identified (Ryan et al., 2021). Almost 20 species of them have been observed in humans, with *Cryptosporidium hominis* (*C. hominis*) and *Cryptosporidium parvum* (*C. parvum*) being the two most notable species (Innes et al., 2020).

In the first 2 years of life, *Cryptosporidium* sp. is the second most prevalent cause of diarrhea in developing countries (Kotloff et al., 2012). In seven areas of Sub-Saharan Africa and Asia, it is considered the fourth leading cause of diarrhea (Checkley et al., 2015).

With the highest estimated prevalence range of 21–50%, *Cryptosporidium* sp. is the third most often reported pathogenic protist in African nations. In Egypt, the prevalence of *Cryptosporidium* sp. in humans is between 11 and 20% (Ahmed et al., 2022). Egypt led the way in reporting more than one-third (36/122) of waterborne protozoa reports in Africa, with *Cryptosporidium* sp. likely being common in numerous Egyptian water supplies (Ahmed et al., 2018). Despite the availability of several diagnostic procedures for detecting *Cryptosporidium* sp. in feces, food, and water (Ahmed et al., 2018; Ahmed and Karanis, 2018a,b), diagnosis for *Cryptosporidium* sp. is not controlled in Egyptian laboratories (Ahmed et al., 2022) indicating that the prevalence above is greatly underestimated.

Nitazoxanide is the only drug that has been FDA-approved for treating *Cryptosporidium* sp. infection in people with healthy immune systems. However, the efficacy of nitazoxanide in immunocompromised patients is unclear (CDC, 2021; Schneider et al., 2021). The lack of continuous culturing, cumbersome screening procedures for chemotherapeutic treatments, and a scarcity of genetic manipulation tools limits drug and vaccine research against *Cryptosporidium* sp. (Karanis, 2018; Gururajan et al., 2021).

Severe cases frequently necessitate hospitalization in immunocompetent individuals and are more common in youngsters and the elderly (Shaposhnik et al., 2019; Collier et al., 2021). Immuno-compromised people, including transplant patients, HIV and cancer patients receiving chemotherapy, frequently have to temporarily halt their treatment regimens when they contract *Cryptosporidium* sp. infection (Sparks et al., 2015; Schneider et al., 2021; Ahmed et al., 2023). Significant economic losses and zoonotic relevance are associated with cryptosporidiosis, specifically *C. parvum*. Due to the disease’s harmfulness and the ease with which newborn calves might develop it, it is difficult to control (Zhang et al., 2018). Anti-cryptosporidials would undoubtedly benefit patients at high risk of *Cryptosporidium* infection and the veterinary industry.

Herbal formulations with anti-*Cryptosporidium* sp. activity have been identified in recent years. They proposed effective treatment against *C. parvum in vitro* (Jin et al., 2019; Mendonça et al., 2021). The biodiversity of plants makes them a commonly explored source of novel bioactive compounds, providing molecules with distinct structures, complex or simple, with colossal chemical variety (Morais et al., 2020). Natural products, particularly crude forms, have been used traditionally to treat various parasitic diseases, such as babesiosis, leishmaniasis, malaria, and trypanosomiasis (Ismail et al., 2020; Kingston and Cassera, 2022). Moreover, natural products have afforded the pharmaceutical industry potential antiparasitic drugs, such as the antimalarial drugs artemisinin from *Artemisia annua* and quinine from *Cinchona succirubra* (Kayser et al., 2002) in addition to licochalcone A, amphotericin B, and ivermectin (Kayser et al., 2003).

Asteraceae (Compositae) is considered one of the most prominent and widely distributed plant families (Panda and Luyten, 2018). In Egypt, this plant family comprises 14 genera, including the genus *Artemisia* (Mokhtar et al., 2019) which is represented by five species; *A. monosperma* Delile*, A. vulgaris, A. verlotiorum, A. scoparia* Waldst and *A. judaica* L. (Badr et al., 2012). Plants of this genus were reported to possess antioxidant, antimicrobial, and cytotoxic activities (Bora and Sharma, 2011; Pandey and Singh, 2017). These biological activities are linked to their phytoconstituents, of which terpenoids and phenolics predominate (Bora and Sharma, 2011; Pandey and Singh, 2017; Goda et al., 2021).

*A. judaica* (Asteraceae) is a perennial aromatic shrub, wildly distributed in Egypt, particularly in Sinai, Gabal Elba, Mediterranean, and Red Sea regions (Ahmed et al., 2017) and has been extensively employed in traditional medicine practices. Therefore, it is almost ubiquitous in Egyptian households. *A. judaica* has demonstrated potent wound healing, antiviral, antibacterial, antihelminth, and antiprotozoal properties in previous research (Mokhtar et al., 2019; Kshirsagar and Rao, 2021; EDA, 2022; Marakhova and Emam, 2022; Mohammed et al., 2022; Qanash et al., 2023). Due to its availability, affordability, and anti-protozoal activity, *A. judaica* is a prime candidate for testing against *Cryptosporidium* oocysts. This study, therefore, aims to investigate the cytotoxic activity of *A. judaica* L. ethanolic extract and its fractions against *C. parvum* using (a) *in vitro* experiments, (b) acting mechanism, (c) *in vivo* bioassay to demonstrate the infectivity of the treated oocysts.

## 2. Materials and methods

### 2.1. Oocysts source and staining

Fecal samples containing *Cryptosporidium* sp. oocysts were collected from a farm with a known history of cryptosporidiosis in its newborn calves (Ahmed et al., 2019). The feces of 15 neonatal calves were collected in plastic bags and labeled with the number of calves and their section. The samples were promptly transferred to the Medical Parasitology laboratory. Each sample was smeared directly and allowed to dry at ambient temperature. The samples were fixed and stained using the modified technique of Ziehl-Neelsen procedure (mZN) (Henriksen and Pohlenz, 1981) to determine the infection status and oocysts load. Four samples with significant cryptosporidial infection (≥ 10–20 oocysts/field) were chosen for additional processing in the experiment.

Approximately 2 mL of the watery fecal specimens were immediately transferred into 2-mL Eppendorf tubes and stored at minus 20°C for further molecular identification and confirmation. The remaining part of fecal samples containing oocysts were filtered through four layers of gauze and stored in potassium dichromate (K_2_Cr_2_O_7_) (1:4, *v/v*) for subsequent oocysts processing at 4°C.

Before purification, the suspension of preserved oocysts and potassium dichromate was concentrated using the formalin-ethyl acetate technique, but potassium dichromate was used in place of formalin. Briefly, after thorough mixing, the potassium dichromate fecal suspension was split into 15 mL Falcon tubes. The top three milliliters of each tube were produced by adding ethyl acetate to the fecal - potassium dichromate suspension. Before centrifuging for 10 min at 2100 g, the tubes were tightly sealed and shook for 20 s. Using a wooden stick, the top lipid layer was discarded after being loosened. After decanting the supernatant, the residual sediment was washed three times with phosphate-buffered saline (PBS) (0.324 g KH_2_PO_4_, 1.368 g Na_2_HPO_4_, 7.014 g NaCl, 0.651 g KCl, and 1,000 mL H_2_O; pH 7.4) (Arrowood and Sterling, 1987).

Each sample’s 3 mL final volume of concentrated sediment was mixed in potassium dichromate at 4°C until the purification process commenced.

#### 2.1.1. Purification of *Cryptosporidium* oocysts

To purify oocysts from previously concentrated fecal samples, discontinuous gradient sucrose flotation was applied (Castro-Hermida et al., 2004; Kourenti and Karanis, 2006). On a discontinuous gradient of sucrose, potassium dichromate-preserved sediments were layered.

Solution A (1:2 v/v, specific gravity 1.11) and solution B (1:4 v/v, specific gravity 1.07) were prepared by diluting a stock solution of modified Sheather’s sugar (El-Nasr Co. for Intermediate Chemicals, Egypt) (500 g sucrose, 320 mL water, and 6.5 g phenol) with PBS. In a 15-mL conical tube, 5 mL of solution A was poured first, followed by 5 mL of solution B.

Oocysts were previously condensed into a suspension (3 mL), which was then carefully poured over the gradients. For 30 min, the gradient tubes were centrifuged at 1200 g (Universal Centrifuge, PLC-012E). After the second layer from the top had been separated, it was washed in saline and finally PBS. Decanting the supernatant allowed the sediments (about 1 mL/tube) to be examined in wet mount (WM) and mZN stain for oocysts of *Cryptosporidium* sp. Purified oocysts were stored in a solution of 2.5% potassium dichromate 1:4 v/v at 4°C for use in subsequent research within 4 months period.

#### 2.1.2. Molecular identification of *Cryptosporidium* species

The species/genotypes of *Cryptosporidium* used were determined using molecular identification. Frozen fecal samples were thawed in cold PBS and filtered through two layers of gauze. PBS washed and centrifuged at least three times. The Qiagen DNA stool-mini kit were used to extract DNA from 200 μL of collected silt, per manufacturer directions (50, manufactured in Germany). The Qiagen method was modified by raising the water bath temperature to 90°C and storing a 100 μL DNA aliquot at – 20°C for PCR amplification.

Nested PCR primers CPr I (5’-AAACCCCTTTACAAGTATCAATTGGA-3′) and CPr II (5’-TTCCTATGTCTGGACCTGGTGAGTT-3′), CPr III (5’-TGCTTAAAGCAGGCATATGCCTTGAA-3′) and CPr IV (5’-AACCTCCAATCTCTAGTTGGCATAGT-3′) were employed to amplify the small subunit ribosomal RNA gene of the four calves’ fecal samples (Bialek et al., 2002). With previously mentioned modification (Ahmed et al., 2019), initial amplification was conducted at 94°C for 5 min, followed by 35 cycles of 94°C for 30 s, 55°C for 30 s, and 72°C for 45 s, with the final extension at 72°C for 5 min. After electrophoresis on a 1% agarose gel stained with ethidium bromide, the amplified products were visualized using a UV transilluminator.

### 2.2. Phytochemical investigations of *Artemisia judaica* L.

#### 2.2.1. Plant collection and preparation of its crude extract

**Figure 1:**
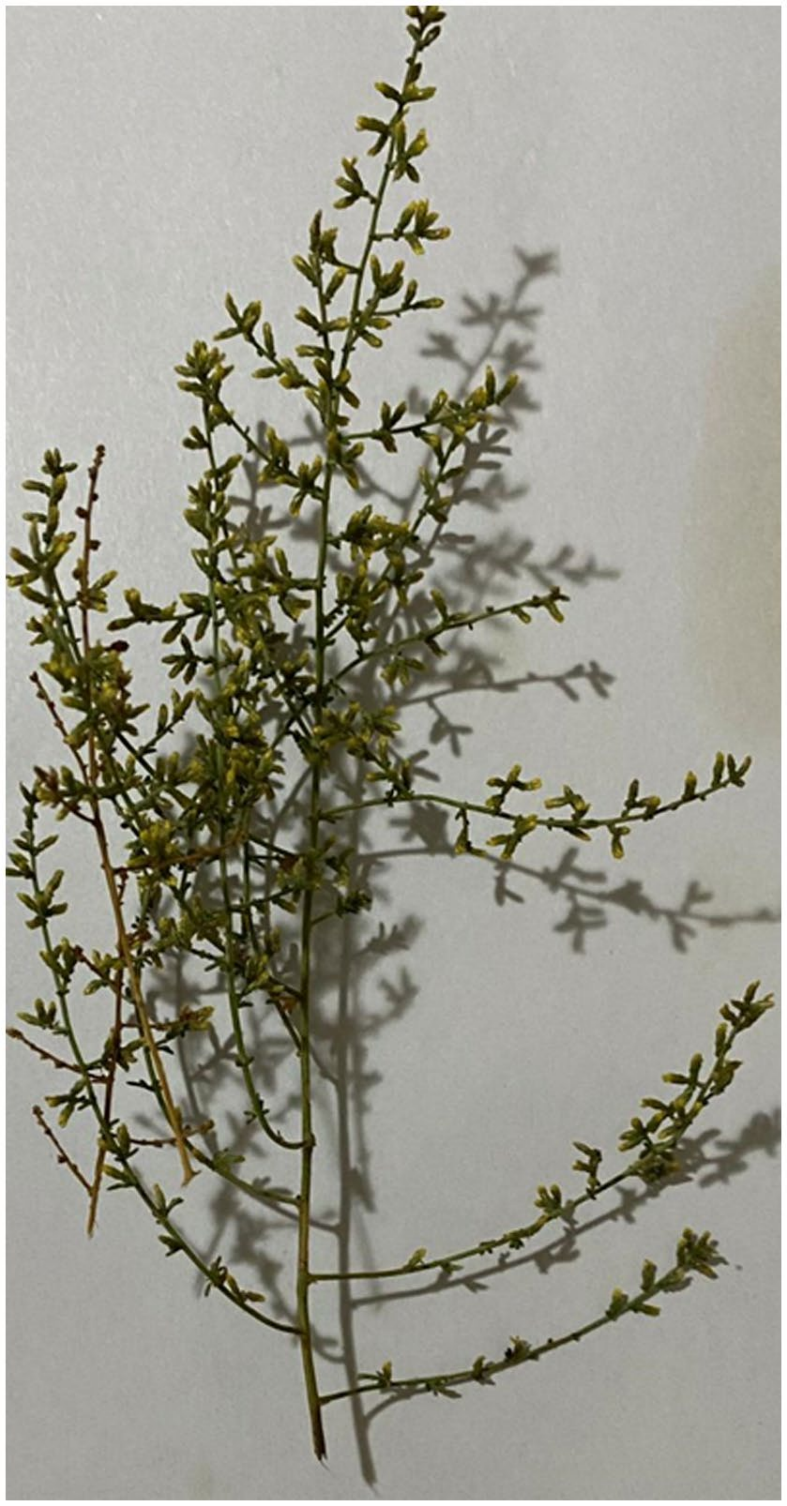
The aerial parts of *Artemisia judaica* L.

*A. judaica* aerial parts (Figure 1) were bought from the Egyptian market in September 2018. Plant authentication was carried out by Prof. Dr. Elsayeda M. Gamal El-Din (Department of Botany, Faculty of Science, Suez Canal University). A voucher sample of the plant (#Aj-2018-1) was placed in the herbarium of Pharmacognosy Department, faculty of Pharmacy, Suez Canal University.

Aerial parts of *A. judaica* were air-dried at room temperature in shade for 1 week prior to grinding. As previously mentioned, (Mokhtar et al., 2019), the crude ethanolic extract of *A. judaica* (ArEx) was prepared by macerating 0.2 Kg of the plant twice with 95% ethyl alcohol (300 mL, 48 h) at 25°C. The combined extracts were vacuum - concentrated to obtain *A. judaica* crude extract (17 g), which was then kept at 4°C.

#### 2.2.2. Preparation of terpenoids and phenolic fractions of *Artemisia judaica* L.

The terpenoid fraction of *A. judaica* was prepared according to a previously described method (Leyva-López et al., 2016) with few modifications. The crude *A. judaica* extract (5 g) was extracted with chloroform (3 × 25 mL). The chloroform extracts were filtered, combined then concentrated under reduced pressure to afford 0.45 g of the terpenoid fraction of *A. judaica* (ArT).

The phenolic fraction of *A. judaica* was prepared according to a previously mentioned method (Eltamany et al., 2022). An amount of 10 g of *A. judaica* crude extract was suspended in aqueous solution of 5% Na_2_CO_3_ before being extracted chloroform (3 × 50 mL). The aqueous extract lift was then acidified with HCl and fractionated successively with chloroform, ethyl acetate, and *n*-butanol. These obtained extracts were combined and vacuum dried to yield approximately 8.5 g of the phenolic fraction of *A. judaica* (ArPh).

#### 2.2.3. Estimation of total phenolic and total flavonoids contents in ArEx

Total phenolics content (TPC) in ArEx was quantified spectrophotometrically by the Folin–Ciocalteu assay as previously described (Eltamany et al., 2020, 2022) where gallic acid was served as a standard. At λ 630 nm, the UV absorbances against blank were recorded in triplicate by Milton Roy Spectronic 1,201 UV/Vis Spectrophotometer (Houston, TX, United States). The result was obtained in terms of gallic acid equivalents (mg·GAE/g dry extract).

The total flavonoid content (TFC) was quantified by AlCl_3_ method, as previously stated (Eltamany et al., 2020). Quercetin was employed as a standard. The UV absorbances against blank at λ 510 nm were measured in triplicate using Milton Roy Spectronic 1,201 UV/Vis Spectrophotometer (Houston, TX, United States). The result was expressed as quercetin equivalent per gram of dry extract (mg QE/g).

#### 2.2.4. Estimation of total terpenoids content in ArEx

The total terpenoids content (TTC) in ArEx was analyzed quantitatively using Salkowski test described by Kim et al. (2018). Linalool (a monoterpene compound) was employed as a standard. The absorbance at λ538 nm against blank was recorded in triplicate by Milton Roy Spectronic 1,201 UV/Vis Spectrophotometer (Houston, TX, United States). TTC of *A. judaica* was determined as linalool equivalents per gram of dry extract (mg Linol/g).

### 2.3. *In vitro* studies of antioxidant activity and cytotoxic assays of *Artemisia judaica* L against *Cryptosporidium* oocysts

#### 2.3.1. Evaluation of the *in vitro* antioxidant activity of *Artemisia judaica* L.

##### 2.3.1.1. DPPH free radical scavenging activity of *Artemisia judaica*

The ArEx as well as its ArPh and ArT fractions were investigated for their scavenging activity of 2,2-diphenyl-1-picrylhydrazyl (DPPH) radical using the procedure previously detailed (Eltamany et al., 2020, 2022). Ascorbic acid served as a standard.

In triplicate, the absorbances at λ 515 nm were recorded against blank by Milton Roy Spectronic 1,201 UV/Vis Spectrophotometer (Houston, TX, United States). The DPPH radical quenching the sample of was calculated applying this equation: PI = [{(AC − AT)/AC} × 100].

Where PI = percent inhibition, AC = control absorbance and AT = absorbance of DPPH + sample mixture. The IC_50_ of DPPH was estimated from the dose/response curve created by Graphpad Prism software (San Diego, CA, United States).

##### 2.3.1.2. ABT assay of *Artemisia judaica*

The ability of ArEx, ArT, and ArPh to capture and neutralize ABTS^•+^ radical cation [2, 2′-azino-bis (3-ethylbenzothiazoline-6-sulfonic acid)] was assayed using the procedure described in detail by Abouseadaa et al. (2020). Butylhydroxytoluene (BHT) was used as a positive control. The absorbances at λ 734 nm were recorded in triplicate against blank by Milton Roy Spectronic 1,201 UV/Vis Spectrophotometer (Houston, TX, USA).

The ABTS^•+^ neutralizing activity of a sample was estimated using the following equation: PI = 100 [(Control – Sample)/Control].

The IC_50_ was obtained from the dose/response curve obtained by Graphpad Prism software (San Diego, CA, United States).

##### 2.3.1.3. Ferric reducing antioxidant power assay for *Artemisia judaica*

The reducing power of ArEx, ArPh, and ArT was estimated spectrophotometrically by FRAP assay mentioned before (Eltamany et al., 2020, 2022). This assay is based on the reduction of ferricyanide ions in to ferrocyanide by an antioxidant substance.

At λ700 nm, the absorbances were measured against a blank (in triplicate) by Milton Roy Spectronic 1,201 UV/Vis Spectrophotometer (Houston, TX, USA). Ascorbic acid and BHT were utilized as standards. Results were represented in terms of m Mol Fe^+2^ equivalents per gram of dry sample (mMol Fe^+2^/g).

##### 2.3.1.4. Total antioxidant capacity assay for *Artemisia judaica*

TAC of ArEx, ArT, and ArPh were estimated using the phosphomolybdenum spectrophotometric assay according to the method previously described (Eltamany et al., 2020, 2022). This test is based on the conversion of Mo^+6^ to Mo^+5^ in an acidic media by an antioxidant substance, resulting in a green phosphate/Mo^+5^ complex.

The absorbances were recorded in triplicate at λ 695 nm against blank by Milton Roy Spectronic 1,201 UV/Vis Spectrophotometer (Houston, TX, USA). The standards were ascorbic acid and BHT. Data were obtained as mg equivalents of gallic per gram of dry extract (mg GAE/g).

#### 2.3.2. Cytotoxic assay of *Artemisia judaica* against *Cryptosporidium* oocysts using MTT assay

The MTT assay was performed to primarily determine the most potent herbal treatment of *A. judaica* L (ArEx, ArPh, and ArT) against *C. parvum* oocysts. In a 96-well plate, *C. parvum* oocysts-PBS suspension were plated in triplicate at a density of 1 × 10^4^ oocysts. The oocysts were treated with ArEx, ArPh, and ArT at concentrations of (100, 200, 500, 1,000, and 2000 μg/mL). These concentrations were produced in a double-fold manner. Oocysts viability was assessed after 48 h using the MTT assay kit “Promega, New York, NY, USA” (Mosmann, 1983). Three hours after adding MTT dye (3-(4,5-dimethylthiazol-2-yl)-2,5-diphenyl-2H-tetrazolium bromide) to the wells of a microtiter plate, the plate was incubated at 37°C.

Using an ELISA microplate reader, the absorbance was determined to be 570 nm (BIO-RAD, model iMark, Tokyo, Japan). Half-maximal inhibitory concentration (IC_50_) values were computed from the relative viability using GraphPad prism 7. A blank treatment of PBS and ethanol was applied to three wells, and untreated *C. parvum* oocysts were utilized as a control in another three wells. Based on the results of MTT assay, ArPh was used in the subsequent experiments.

#### 2.3.3. Parasitological toxic activity of ArPh against *Cryptosporidium parvum* oocysts

##### 2.3.3.1. *In vitro* exposure of *Cryptosporidium parvum* oocysts to ArPh

Within 2 weeks of preparation of the previously prepared suspension (section 2.1.1), the concentration of cryptosporidial oocysts was determined using a Neubauer hemocytometer. After multiple washing with PBS (to remove potassium dichromate), the stock suspension was diluted by a factor determined by the mean of four hemocytometer counts (Finch et al., 1993; Karanis and Schoenen, 2001).

Between the hemocytometer cover slide and counting chambers, oocyst suspension in a 10-μL aliquot was pipetted. Counting oocysts were performed under a 400x dry microscopic objective. A final concentration of oocysts/PBS suspension (1 × 10^5^/mL) was stored. An antibiotic suspension was added at room temperature (10,000 U Pen/ml, 10,000 g Strep/ml, and 25 g Amphotericin B/mL).

To generate a total volume of 1 mL, various quantities of ArPh (100, 200, 500, 1000, 2000 μg/mL) were added to the estimated oocysts solution. A control of untreated oocysts (*C. parvum* oocysts, PBS, antibiotic) was used for comparison with ArPh-treated oocysts (ArPh-TO). Assessments of the cytotoxic effects of ArPh on oocysts’ number, viability, and shape were made after 2, 24, and 48 h of incubation. For each time period, the findings were compared to a control group, and the differences between the two groups were computed.

With the aid of a viability dye [0.4% Trypan Blue (TB)], the number of oocysts/μL was calculated after measuring the oocysts distress in count on a Neubauer hemocytometer of Kao and Ungar (1994).

The lethal dose was calculated by applying the destruction rate equation (A-B/A) and multiplying it by 100. In this equation, A represents the mean number of intact oocysts found in the control tube, and B represents the mean number of intact oocysts found in the ArPh-TO tube (Ahmed et al., 2019).

Alterations in morphological size were captured on camera with the use of an Olympus bright field 1,000x oil immersion lens and an ocular micrometer. The *in vitro* exposure experiment was carried out with three separate replicates for each different concentration of ArPh. Based on preliminary trials, the different ArPh concentrations were chosen (Ahmed and Mokhtar, unpublished data).

##### 2.3.3.2. *In vitro* evaluation of the effect of ArPh on *Cryptosporidium parvum* oocysts using the comet assay

The neutral comet assay was applied to *C. parvum* oocysts exposed to varying doses of ArPh based on Ostling and Johanson (1984). ArPh-TO was mixed with molten low-melting-point agarose at a ratio of 1:10 (*v/v*), and 75 μL of this mixture was immediately imbedded in a CometSlide (frosted slide) placed flat at 4°C in the dark for 10 min.

The slides were then placed in lysis buffer (2.5 M NaCl, 100 mM NaEDTA, 10 mM Tris pH 10, 1% Triton X, and 10% dimethylsulphoxide (DMSO; Sigma) at pH 10) with the coverslips removed. To remove the lysis solution from the slide, it was placed twice for 5 min at room temperature in electrophoresis buffer (90 mM Tris base, 90 mM boric acid, and 2 mM ethylenediaminetetraacetic acid). The slide was then placed in a horizontal electrophoresis tank, and 20 min of electrophoresis at 28 V (1 V/cm, 400 mA) was conducted. After removing the excess electrophoresis buffer from the slide, it was gently rinsed in distilled water. The gel was then fixed by immersion in 70% ethanol for 5 min and air-dried for 15 min at room temperature. The slide was stained with 50 μL of 20 μg/mL ethidium bromide for 1 h. The migrating DNA (comet) was spotted at 400x magnification using an Epifluorescence optika microscope.

Using an automatic image analysis system (Comet score V software), DNA damage was determined by measuring the tail moment, which is defined as the product of the amount of DNA in the tail and the displacement between the center of mass of the comet head and the center of mass of the tail (tail length) (Olive and Banáth, 2006). The olive tail moment was defined as tail DNA% multiplied by tail moment (Cell Biolab, 2013). Each experimental group received triplicate samples, from which 100 oocysts were randomly selected and analyzed. As a control, the same previous technique was used to handle DNA damage of *C. parvum* untreated oocysts in PBS.

### 2.4. *In vivo* experiment (infectivity assay)

#### 2.4.1. Animals

Nine-week-old male SPF Swiss Albino mice weighing an average of 24–32 g were purchased from The Egyptian Organization for Biological Products and Vaccines (Vacsera, Cairo, Egypt), and maintained under a regular day/night cycle and sanitary conditions. The animals were pathogen-free, as evidenced by a sanitary certificate. Before the experiment, the mice were acclimatized to the experimental environment for 10 days and basal diet and water were administered *ad libitum*. Even though the mice were guaranteed to be free of pathogens when they were acquired, WM and mZN were used to check their feces for three successive days (Ma and Soave, 1983; Ungar et al., 1990).

#### 2.4.2. Study design

The mice were randomly divided into seven groups of three mice each to conduct a biological test to determine the infectivity of treated *C. parvum* oocysts (Karanis and Schoenen, 2001).

Group 1: Negative control (non-infected animals, untreated).

Group 2: Positive control (infected and untreated animals).

Groups 3–7: Infected animals with the five concentrations of ArPh-TO.

In the animal house of the Faculty of Medicine at Suez Canal University, every mouse was kept individually and in accordance with the established protocols for animal care.

#### 2.4.3. Infectivity investigation

Using a hemocytometer/TB, 500 μL of suspension inoculums were administered to the mice 48 h after treatment (section 2.3.3.1) (Karanis and Schoenen, 2001). All the mice were administered the estimated inoculums via gavage at the same time. Each mouse’s fresh feces were collected by exerting moderate pressure to their abdomens. Purified fecal samples from mice were examined post-inoculation for oocysts shedding at third, fifth, six, seventh and 10 days using mZN stain. On the tenth day after inoculation, all the animals were killed by dislocating the cervix. The infection status of each group was determined based on microscopic observations of *Cryptosporidium* oocysts and was graded as positive or negative (Korich et al., 2000).

### 2.5. Statistical analysis

Three independent experiments with three replicates per test sample were used to conduct the MTT assay. The mean absorbance values were represented as a percentage of the values of the untreated control oocysts standard error of the mean. The IC_50_ was expressed with a non-linear regression curve fit using Microsoft Excel software. The statistical significance was calculated using an unpaired t-test. One-way analysis of variance (ANOVA) test was used to determine the statistical significance of differences between treatments and the control group in the hemocytometer and comet assays. The *p* values of ≤ 0.05 were considered significant. IBM SPSS Statistics V26.0 was used for all statistical analyzes (IBM Corp., Armonk, NY, United States). The charts were made with GraphPad Prism 7. (Dotmatics, San Diego, CA, United States).

## 3. Results

### 3.1. Genetic identification of *Cryptosporidium* oocysts by nested PCR assay

**Figure 2:**
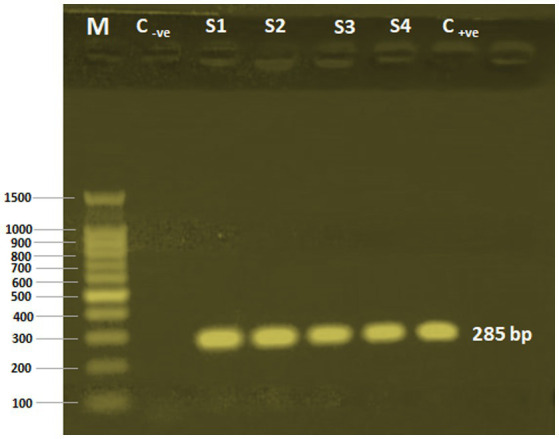
PCR-nested analysis of four calves’ fecal samples. M, 100 bp DNA ladder (Applied Biotechnology Co. Ltd., Egypt); C-ve, Control negative; S1-S4: *Cryptosporidium* oocysts sample sources; C+ve, Control positive.

The *Cryptosporidium* species in the examined samples was determined using a *C. parvum*-specific nested PCR technique. *C. parvum* was present in all the calves’ fecal samples. The four employed samples’ amplified products of 285 bp were successfully electrophoresed (Figure 2).

### 3.2. Phytochemical investigations of *Artemisia judaica* L.

#### 3.2.1. Total phenolic, flavonoids, and terpenoids contents

Using the Folin–Ciocalteu colorimetric technique, the TPC of ArEx was quantified to be 52.6 ± 3.1 mg GAE/g extract. While the TFC of ArEx was estimated to be 64.5 ± 3.1 mgQE/g of plant extract using AlCl_3_ spectrophotometric method (Table 1).

**Table 1:** TPC, TFC and TTC of *A. judaica* crude extract.

Sample	TPC (mg GAE/g)	TFC (mgQE/g)	TTC (mg Linol/g)
ArEx	52.6 ± 3.1	64.5 ± 3.1	9.5 ± 1.1

Data are expressed as mean ± SD of three independent values. TPC, Total phenolic content; TFC, total flavonoids content; TTC, Total terpenoids content; GAE, Gallic acid equivalents; ArEx, *A. judaica* ethanolic extract.

The TTC of ArEx was quantified spectrophotometrically at λ538 nm based on the intensity of the reddish-brown color produced by them. As depicted in Table 1, the TTC of ArEx was found to be 9.5 ± 1.1 mg Linol/g.

#### 3.2.2. Evaluation of antioxidant activity of ArEx, ArT, ArPh

As shown in Table 2, among ArEx, ArT, and ArPh, the ArPh exhibited a distinctive radical scavenging ability on DPPH and ABT with IC_50_ = 47.27 ± 1.86 μg/mL and 66.89 ± 1.94 μg/mL compared to ascorbic acid and BHT (IC_50_ = 10.64 ± 0.82 μg/mL and 141.98 ± 3.46 μg/mL), respectively.

**Table 2:** Antioxidant activities of ArEx, ArPh, and ArT by DPPH, ABT, FRAP and TAC assays.

Sample	DPPH (IC_50_ in μg/mL)	ABT (IC_50_ in μg/mL)	FRAP (mMol Fe^+2^/g)	TAC (mg GAE/g)
ArEx	123.83 ± 2.71	154.15 ± 4.92	2.34 ± 0.42	40.91 ± 2.63
ArT	233.24 ± 5.17	481.92 ± 8.76	1.38 ± 0.26	23.54 ± 1.98
ArPh	47.27 ± 1.86	66.89 ± 1.94	2.97 ± 0.65	46.23 ± 3.15
Ascorbic acid	10.64 ± 0.82	NT	7.41 ± 0.57	72.68 ± 3.74
BHT	NT	141.98 ± 3.46	2.86 ± 0.38	79.31 ± 3.95

Data expressed as mean ± SD of three independent values. NT, Not tested. ArEx, *A. judaica* ethanolic extract; ArPh, *A. judaica* phenolic fraction; ArT, *A. judaica* terpenoid fraction; DPPH, 2,2-diphenyl-1-picrylhydrazyl; FRAP, Ferric reducing antioxidant power; TAC, Total antioxidant capacity; Fe^+2^, Iron; GAE, Gallic acid equivalents; BHT, Butylated hydroxytoluene.

The ferric ion reducing power of ArEx, ArT, and ArPh expressed in terms of mMol Fe^+2^ /g were demonstrated in Table 2. The ArPh possessed the highest reducing power 2.97 ± 0.65 mMol Fe^+2^ /g, compared with ArEx (2.34 ± 0.42 mMol Fe^+2^ /g) and the ArT (1.38 ± 0.26 mMol Fe^+2^ /g). Ascorbic acid (7.41 ± 0.57 mMol Fe^+2^ /g) and BHT (2.86 ± 0.38 mMol Fe^+2^ /g) served as positive controls.

The obtained results (Table 2) revealed that the ArPh exhibited the highest TAC (46.23 ± 3.15 mg GAE/g) in comparison with the ArEx (40.91 ± 2.63 mg GAE/g) and the ArT (23.54 ± 1.98 mg GAE/g). The TAC of the positive controls, ascorbic acid and BHT, were 72.68 ± 3.74 mg GAE/g and 79.31 ± 3.95 mg GAE/g, respectively.

### 3.3. *In vitro* oocysts-toxic activity of *Artemisia judaica* L.

#### 3.3.1. Cytotoxicity of ArEx, ArPh, and ArT against *Cryptosporidium* oocysts

**Figure 3:**
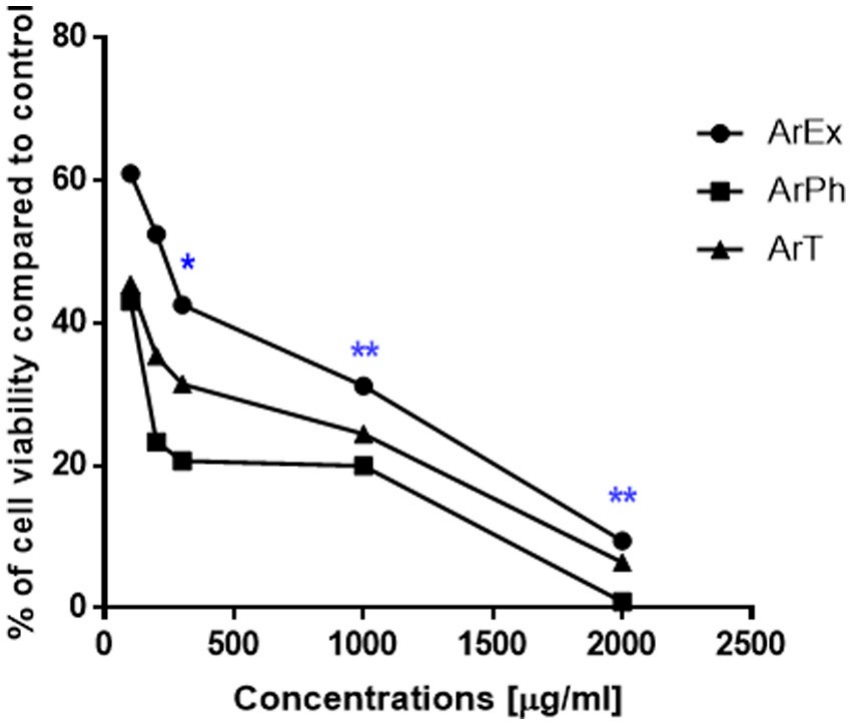
Dose-response curve for the viability at different working concentrations of ArEx, ArPh, ArT using MTT assay. *(*p* ≤ 0.05) and **(*p* ≤ 0.001) significantly different using unpaired *t*-test of treated oocysts and control using GraphPad prism software. ArEx, *A. judaica* ethanolic extract; ArPh, *A. judaica* phenolic fraction; ArT, *A. judaica* terpenoid fraction.

Table 3 summarizes the IC_50_ values of the ArEx, ArPh, and ArT. The ArPh displayed significant toxicity against *C. parvum* oocysts, with an IC_50_ value of 31.6 μg/mL (Table 3) and a cell viability percentage of 0.87% at the highest concentration. In the meantime, ArT displayed promising cytotoxicity with an IC_50_ value of 64.5 μg/mL, and the greatest concentration achieved 6.4% cell viability. With an IC_50_ value of 213 μg/mL and a cell survival rate of 9.44%, the ArEx displayed poor cytotoxicity. The most potent oocysts-toxic compound is ArPh, according to the dose–response curves of ArEx, ArPh, and ArT (Figure 3). Due to its potential oocysts-killing properties, ArPh was selected to be used in subsequent studies.

**Table 3:** IC_50_ values of ArEx, ArPh and ArT using MTT assay against *Cryptosporidium* oocysts.

Sample	Working concentration	^*^IC_50_ [μg/mL]
ArEx	100, 200, 500, 1,000, 2000 μg/mL	213 ± 4.26
ArPh		31.6 ± 1.98
ArT		64.56 ± 2.42

* IC_50_ were expressed as Mean ± SD of three independent trials and calculated by non-linear regression curve fit using EXCEl. ArEx, *A. judaica* ethanolic extract; ArPh, *A. judaica* phenolic fraction; ArT, *A. judaica* terpenoid fraction.

#### 3.3.2. Parasitological *in vitro* exposure of *Cryptosporidium* oocysts to ArPh

##### 3.3.2.1. Activity of ArPh on the number of oocysts

**Figure 4:**
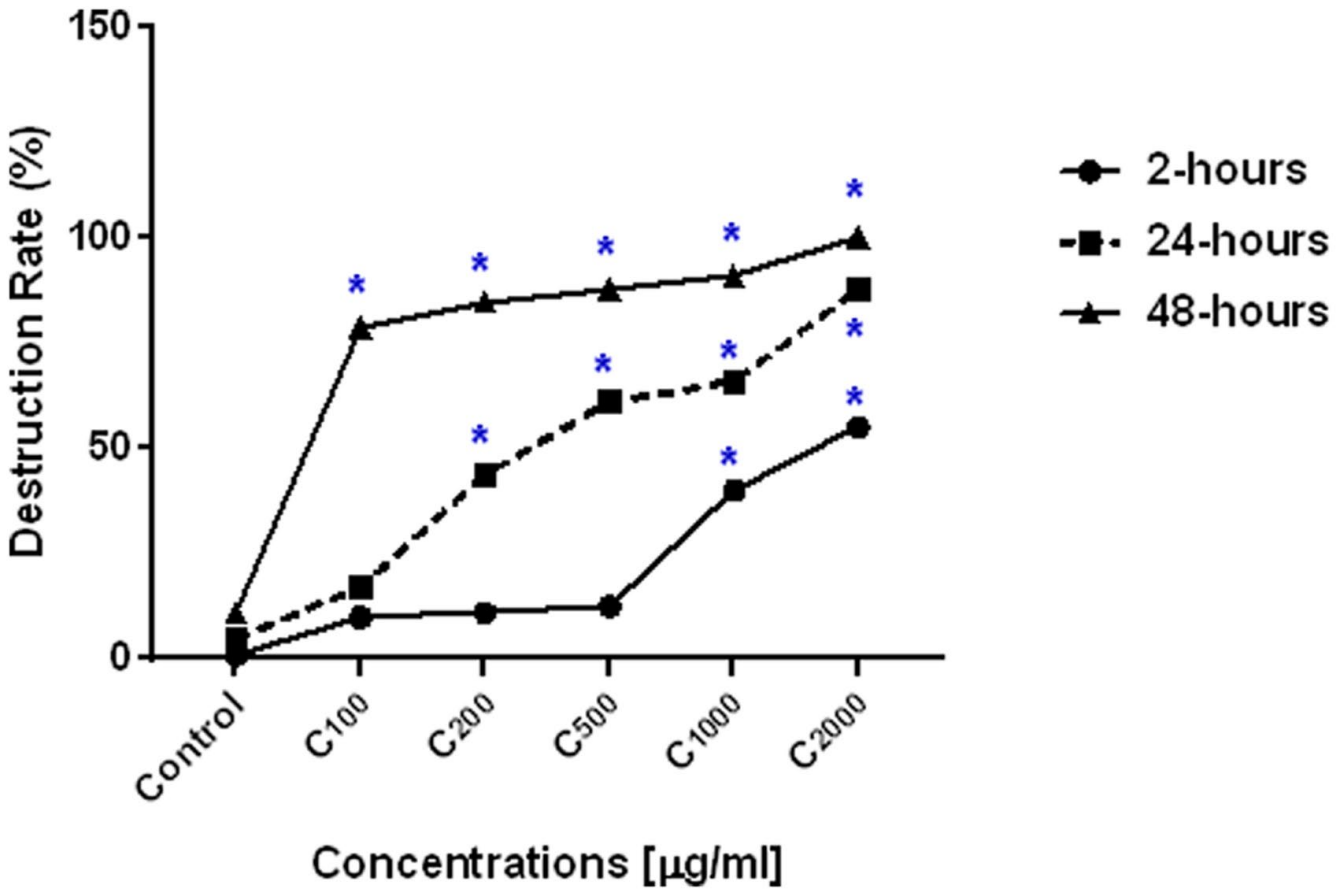
ArPh activity on the number of oocysts. Count distress of oocysts by destruction rate (%) = (A-B/A) x100 in varied periods of exposure with various concentrations of ArPh. The values are presented as the means of three separate experiments. ArPh, *A. judaica* phenolic fraction. *Significant difference between concentrations using one way ANOVA test.

Figure 4; Table 4 depict a summary of differences in ArPh dose-dependent inhibition of *C. parvum* oocysts *in vitro*. Different concentrations of ArPh (100, 200, 500, 1,000, 2000 μg/mL) caused “visible stress” on the total number of *C. parvum* oocysts compared to control (*p* < 0.05; Figure 4). The equation for the rate of destruction was used to calculate the proportion of oocysts that were reduced. The minimal lethal concentration at which 90% of oocysts are killed (MLC_90_) was determined to be 1,000 μg/mL. The highest rate of oocysts destruction (100%) was recorded 48 h later at a concentration of 2000 μg/mL. The data revealed a statistically significant difference (p < 0.05) in the concentrations of ArPh measured at various time intervals throughout the experiment.

**Table 4:** Count distress of ArPh-TO according to different concentrations and different times.

ArPh-concentrations	Time					
	2 h		24 h		48 h	
	^*^Mean ± SD	DR (%)	Mean ± SD	DR (%)	Mean ± SD	DR (%)h
C_100 μg/mL_	9 ± 1.4	10.0 ^c^	8.5 ± 0.7	17.0 ^e^	1.75 ± 0.35	78.8 ^e^
C_200 μg/mL_	9.5 ± 0.7	11.0 ^c^	5.75 ± 1.1	43.9 ^d^	1.25 ± 0.35	84.8 ^d^
C_500 μg/mL_	8.75 ± 0.35	12.5 ^c^	4 ± 0.7	61.0 ^c^	1 ± 0	87.9 ^c^
C_1000 μg/mL_	6 ± 0	40.0 ^b^	3 ± 0.7	65.9 ^b^	0.75 ± 0.35	90.9 ^b^
C_2000 μg/mL_	4 ± 1.4	55.0 ^a^	0.5 ± 1.06	87.8 ^a^	0 ± 0	100.0 ^a^

* All mean values are multiplied by 10^4^. Different super script letters in a column mean significant difference between concentrations using one way ANOVA test. DR, Destruction rate; ArPh, *A. judaica* phenolic fraction.

##### 3.3.2.2. Activity of ArPh and Its impact on the oocyst’s morphology.

**Figure 5:**
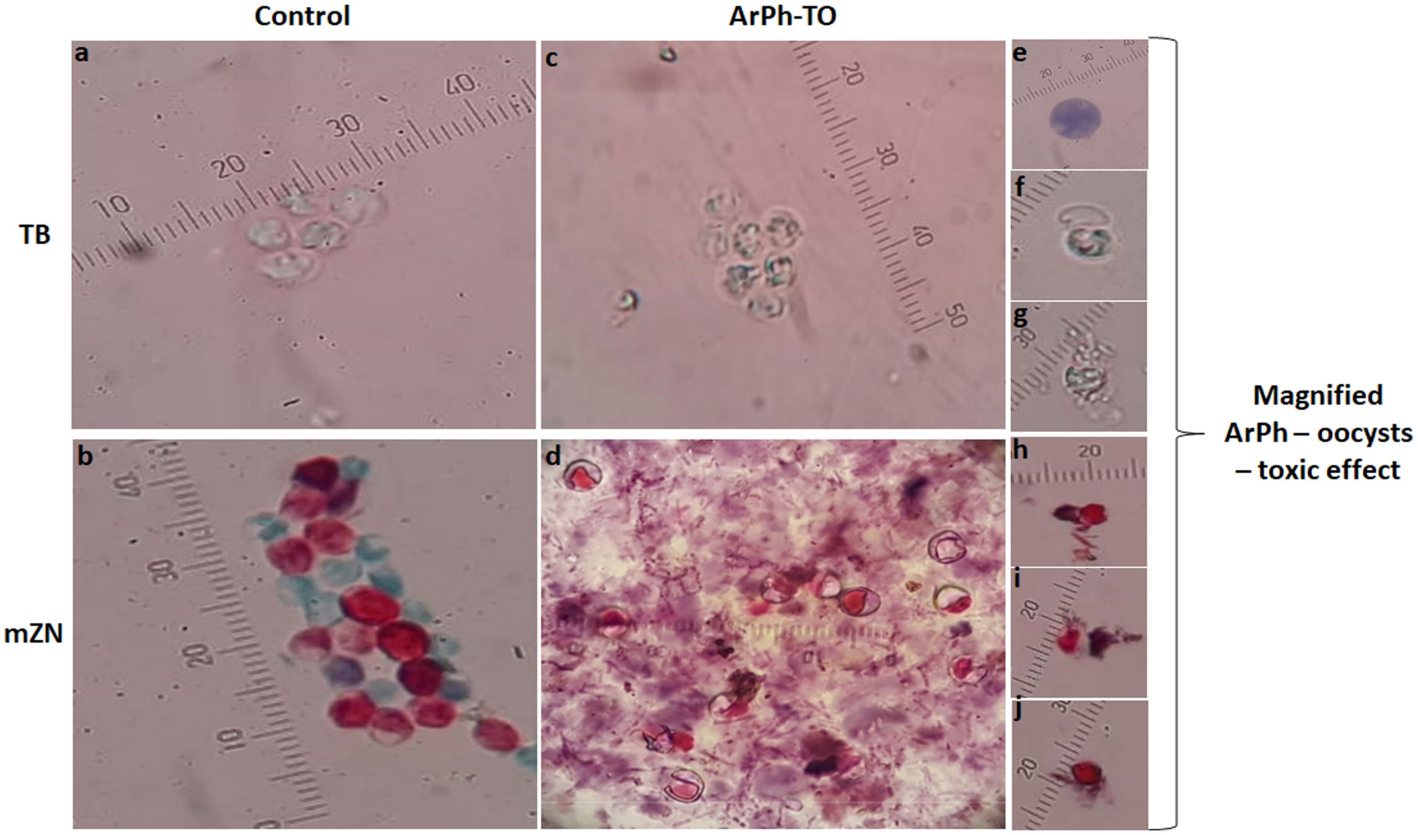
Morphological alterations of ArPh-TO using TB and mZN stains. In the control (vertical arrangement of TB and mZN), the usual architecture of oocysts was displayed photos **(A,B)**. In the ArPh-TO morphological alterations in a group of oocysts were displayed photos **(C,D)**. Cascades of ArPh-TO deaths were magnified and exhibited in single images for TB photos **(E–G)** and mZN photos **(H–J)**. Observable programed oocysts death includes vacuolation, granulation, cracking, content expulsion, and shrinking photos **(E–J)**. A micrometer was added to each photograph to compensate for the varying zoom levels of each shot. ArPh-TO, *A. judaica* phenolic fraction treated oocysts; TB, Trypan blue; mZN, modified Ziehl Neelsen.

Oocysts of *Cryptosporidium* are typically brightly colored, range in size from 4 to 6 micrometers, and consist of a center globule, one to four sporozoites, and eccentric black granules. TB and mZN stain were used to track the morphological changes of oocysts. When ArPh was applied to oocysts at varying concentrations and exposure times, the oocysts exhibited cascades of death beginning with inflating, filling with single/multiple vacuoles, granulation of content, cracking, expulsion of content, and shrinkage (Figure 5). These alterations were first noticed at a concentration of 200 μg/mL and peaked after 48 h with variation among different concentrations.

##### 3.3.2.3. Results of comet assay for ArPh-To.

**Figure 6:**
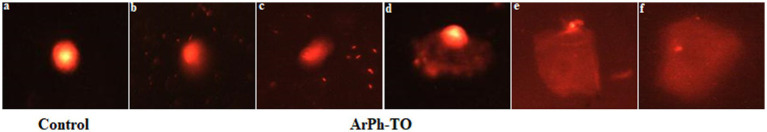
Comet assay indicating changes in nuclear morphology and nuclear DNA fragmentation of *C. parvum* sporozoites when treated with different concentrations of ArPh at the incubation time of 48 hours photos **(B–F)**. The nuclear DNA fragmentation appeared in the DNA tailing, which steadily increased as the fragmentation increased. All stages of nuclear DNA fragmentation photographs **(B–F)** were observed beginning with the concentration of 200 μg/mL. DNA was stained with ethidium bromide and observed by epifluorescence microscopy. Untreated *C. parvum* oocysts was used as a control photo **(A)**.

The nuclear DNA of untreated control *C. parvum* oocysts stained with ethidium bromide was spherical and had a well-defined nucleoid margin (Figure 6A). However, the nuclear DNA of ArPh-TO appeared to be damaged. The nuclear DNA of treated oocysts stained with ethidium bromide looked more prominent and had a longer migratory tail at the different concentrations of ArPh (Figures 6B–F).

In the neutral comet assay, the comet tail moment increased significantly between the control and the different concentrations used with ArPh-TO, in which the tail length and tail DNA (%) increased considerably (*p* ≤ 0.05; Table 5).

**Table 5:** Parameters of the comet assay of *C. parvum* oocysts after exposure to different doses of ArPh.

Samples	% Tailed Mean ± SD	Tail length (PX) Mean ± SD	% DNA in tail Mean ± SD	Tail moment Mean ± SD	Olive tail moment
Control	9.46 ± 0.24 ^f^	8.91 ± 0.08 ^a^	4.97 ± 0.97 ^b^	0.409 ± 0.015 ^b^	8.48 ± 0.01 ^d^
C_100_	15.13 ± 0.40 ^e^	8.06 ± 0.35 ^a^	7.27 ± 0.31 ^a^	0.640 ± 0.03 ^ab^	1.28 ± 0.09 ^ab^
C_200_	17.5 ± 0.28 ^d^	7.09 ± 0.69 ^a^	9.74 ± 0.51 ^a^**	0.705 ± 0.025 ^a^*	1.33 ± 0.06 ^ab^
C_500_	20.4 ± 0.30 ^c^	7.53 ± 1.08 ^a^	8.66 ± 0.45 ^a^	0.783 ± 0.038 ^b^	1.04 ± 0.002 ^c^
C_1000_	24.4 ± 0.48 ^b^	8.36 ± 0.72 ^a^	8.33 ± 0.70 ^a^	0.753 ± 0.13 ^ab^	1.48 ± 0.051^a^***
C_2000_	37.5 ± 0.28 ^a^***	8.44 ± 0.91 ^a^	7.93 ± 0.60 ^a^	0.688 ± 0.11 ^ab^	1.21 ± 0.13 ^bc^

Nuclear DNA damage was determined using an image analysis system. Data are mean ± SD values (*n* = 100). Values with different superscripts in the same column differ significantly at *p* ≤ 0.05. ^***^High significance; ^**^Moderate significance; ^*^Mild significance. Untreated *C. parvum* oocysts were used as a control.

#### 3.3.3. *In vivo* infectivity biological assay of treated oocysts using the mice model

Although the *in vitro* assay demonstrated that at 200 μg/mL oocysts count and morphological changes had begun and at 1000 and 2000 μg/mL ArPh was sufficient to destroy >90% of the visible endogenous components of *C. parvum* oocysts, it is unknown whether the remaining intact oocysts are viable and able to cause infection. Table 6 presents the details and outcomes of the *in vivo* experiment. Infectivity was observed in 2 groups out of 7 (control positive group 2 and treated group 3 with 100 μL ArPh). The rest of the treated groups exhibited no infectiousness (Table 6). Group 1, which served as the experiment’s negative control demonstrated being negative along the duration of the experiment.

**Table 6:** The infectivity assay design and outcomes after the *in vitro* assay of *Cryptosporidium* treated with ArPh.

Code of the group	Mean inoculum per mouse	^*^Inoculation schedule	Ratio of infected mice/total inoculated mice	Starting day of shedding
Group 1	There is no inoculation	500 μL PBS	Zero/Three	None shed oocysts
Group 2	0.925 ×10^5^	500 μL untreated oocysts per animal	Three/Three	d.p.i 3
Group 3	0.175 × 10^5^	500 μL of 100 μg/mL ArPh treated oocysts per animal	One/Three	d.p.i 5
Group 4	0.125 × 10^5^	500 μL of 200 μg/mL ArPh treated oocysts per animal	Zero/Three	None shed oocysts
Group 5	0.1 × 10^5^	500 μL of 500 μg/mL ArPh treated oocysts per animal	Zero/Three	None shed oocysts
Group 6	0.75 ×10^4^	500 μL of 1,000 μg/mL ArPh treated oocysts per animal	Zero/Three	None shed oocysts
Group 7	0 × 10^5^	500 μL of 2000 μg/mL ArPh treated oocysts per animal	Zero/Three	None shed oocysts

^*^Infectivity experiment began on day zero and concluded on day 10 following inoculation. After 48 h of the *in vitro* treatment, mice were given 500 μL of ArPh-TO solution by gastric gavage. Experiments were designed according to Karanis and Schoenen (2001), ArPh-TO, *A. judaica* phenolic fraction treated oocysts; d.p.i., days post inoculation.

## 4. Discussion

Herbals have selective actions against parasites without reduction of host cell viability (Haynes and Krishna, 2004). *Artemisia* was reported worldwide as an effective treatment against several protozoa parasites, including *Giardia duodenalis*, *Blastocystis* sp., *Leishmania major*, and *Plasmodium falciparum* (Mokhtar et al., 2019; Abd-Elhamid et al., 2021; Gruessner and Weathers, 2021; Najm et al., 2021). Since ancient times in Egypt, natural products have been favored and widely used to cure parasitic infections and other diseases (Aboelsoud, 2010). The cost-effectiveness and absence of adverse effects of natural products made them favorable (Ahmed et al., 2019).

*Artemisia judaica* L. is indigenous to Sinai Peninsula (Bakr, 2014). *A. judaica* infusion treats many diseases in folk medicine. Moreover, its diverse pharmacological effects were evidenced (Goda et al., 2021). These bioactivities could be attributed to the plant’s phytochemicals. *A. judaica* accumulates volatile oil, sesquiterpene lactones, and phenolic compounds (Galal et al., 1974; Abdelgaleil et al., 2008; Janaćković et al., 2015). Recent phytochemical research by LC/MS/MS showed that *A. judaica* possessed a plethora of flavonoids, phenolic acids, and terpenoids (Goda et al., 2021).

Plant phenolics are a diverse, widely distributed group of phytochemicals. They comprise stilbenes, tannins, coumarins, phenolic acids and flavonoids (Abotaleb et al., 2019). Polyphenols are natural antioxidants in fruits and vegetables (Abotaleb et al., 2019; Zhang et al., 2022). The most predominant polyphenolic compounds in the plant kingdom are phenolic acids and flavonoids (Chen et al., 2022). In recent years, plant polyphenols have received increasing attention and drawn much interest due to their diverse biological activities (Zhang et al., 2022), including antiprotozoal activities (Phillipson and Wright, 1991). Therefore, In the current study, the total phenolics, flavonoids contents in *A. judaica* were quantified. The obtained results revealed that the plant was a rich source of phenolic and flavonoids (52.6 ± 3.1 mgGAE/g, 64.5 ± 3.1 mg QE/g, respectively).

On the other hand, terpenes and terpenoids are a large, chemically, and functionally diverse group of secondary metabolites, among which several members exhibited cytotoxic, antimicrobial, and antiparasitic effects (Ajikumar et al., 2008). Thus, in this study, the amount of such compounds in *A. judaica* was estimated spectrophotometrically, where linalool was employed as a standard. Our findings demonstrated that *A. judaica* contained a considerable amount of terpenoids (9.5 ± 1.1 mg Linol/g).

Plant antioxidants, among them polyphenolics (phenolic acids and flavonoids) and terpenoids exert their effect through various processes, including metal reduction, chelation, hydrogen transfer, electron transfer, and singlet oxygen quenching (Graßmann, 2005; Eltamany et al., 2020). In the current study, reactive oxygen species (ROS) scavenging mechanisms, the antioxidant potential of the ArEx, as well as ArT and ArPh, was evaluated simultaneously using four indicative assays (DPPH, ABT, FRAP and TAC). The current study findings demonstrated that ArPh exhibited the highest DPPH and ABTS^•+^ radical quenching effects (IC_50_ values of 47.27 ± 1.86 μg/mL and 66.89 ± 1.94 μg/mL, respectively) as well as the FRAP and TAC (2.97 ± 0.65 mMol Fe^+2^/g and 46.23 ± 3.15 mg GAE/g, respectively). The ArEx showed noticeable anti-free radical effects against DPPH and ABTS^•+^ (IC_50_ = 123.83 ± 2.71 μg/mL and 154.15 ± 4.92 μg/mL, respectively), FRAP (2.34 ± 0.42 mMol Fe^+2^/g) and TAC (40.91 ± 2.63 mg GAE/g) compared to ArT (233.24 ± 5.17 μg/mL, 481.92 ± 8.76 μg/mL, 1.38 ± 0.26 mMol Fe^+2^/g and 23.54 ± 1.98 mg GAE/g, respectively).

ROS boost infection as they regulate growth, proliferation, pathogenesis, and virulence of bacteria, viruses, and parasites at physiological levels as cellular signaling molecules (Goes et al., 2016; Szewczyk-Golec et al., 2021). Excessive generation of ROS can generate oxidative stress, resulting in cell damage and death. Consequently, cells include antioxidant networks to scavenge excess ROS (Poljsak et al., 2013). For instance, iron-dependent ROS signaling triggers the differentiation of virulent *Leishmania amazonensis* amastigotes (Mittra and Andrews, 2013). *Blastocystis hominis* virulence and infectivity were increased by oxidant-antioxidant homeostasis changes. Oxidative stress was also crucial to *C. parvum* infection in mice (Bhagat et al., 2017). Antioxidants slow infectious illnesses (Kaur et al., 2018). *In vivo* anti-*Cryptosporidium* actions were reported in olive and fig extracts, which increased glutathione reduced form, superoxide dismutase and catalase plasma levels compared to the infected control group (Abd El-Hamed et al., 2021).

The oocysts of *Cryptosporidium* sp. are known to be small (4–6 μm) and have a low infectious dose (1–10 oocysts); it is reported that they can survive in water for 6–12 months or longer and can produce epidemics even with the drinking of a treated water (Karanis, 2018; Omarova et al., 2018). The adverse effects and growing resistance to the available antiparasitic medicines used to treat *C. parvum* make it difficult for researchers to identify a natural herbal alternative that is nontoxic and easily accessible in sufficient numbers to replace the market-standard drugs (Abdelmaksoud et al., 2020). Therefore, *Cryptosporidium* sp. would be a parasite of particular interest in terms of its interaction with *A. judaica*.

Based on the preceding considerations and the demonstrated antioxidant activity of *A. judaica* crude extracts and fractions; specifically, the phenolic one, the ArEx and its fractions; ArPh and ArT were tested for their toxicity to *C. parvum* oocysts using the MTT assay. This colorimetric test estimates cellular metabolic activity as an indicator of cell viability and cytotoxicity. The ArPh demonstrated a potent toxic effect against *C. parvum* oocysts with an IC_50_ value of (IC_50_ = 31.6 μg / mL). MTT assay is the gold standard for cytotoxicity testing and promptly determines the viability of microorganisms following drug treatment (Iqbal and Keshavarz, 2017; Holzhausen et al., 2019). The potency of phenolic fraction in the present study is aligned with earlier studies that revealed the high concentration of phenolics was highly efficient in the treatment of quinine-resistant malaria (Ferreira et al., 2010; Fordjour and Adjimani, 2020; Mamede et al., 2020).

Since the phenolic fraction of *A. judaica* was the most effective treatment based on the MTT assay, it was chosen to evaluate the effect of ArPh on *C. parvum* oocysts using parasitological experiments. The current investigation indicated that the harmful activity of ArPh against *C. parvum* oocysts is dose-dependent (Table 4). The influence significantly affected the count and the oocysts’s morphological characteristics. Another study proved that six polyphenolic compounds displayed anti - *C. parvum* and anti - *Encephalitozoon intestinalis* action, indicating that these compounds may be employed alone or in conjunction with other moderately active drugs to enhance efficacy (Mead and McNair, 2006). Against *Trypanosoma brucei* and *Toxoplasma gondii* cultures, phenolic compounds displayed strong action (Sun et al., 2016; Jafari et al., 2021). Extracts of the *Artemisia herba-alba* plant included a variety of phenolic compounds, which had antibacterial and antioxidant effects, and hence slowed the growth of both gram-positive and gram-negative bacteria (Mohammed et al., 2021).

In the current study, oocysts stained with TB and mZN exhibited microscopically observable morphological alterations produced by ArPh. The ArPh-TO were lysed, resulting in the loss of oocysts membrane and the expulsion of their contents (Figure 5). ArPh induced morphological alterations in *C. parvum* oocysts due to a cascade of programmed cell death, during which the oocysts membrane became deformed and created pores inside the cell membrane, resulting in cell lysis and death of the parasite. In another study that examined the effect of chitosan nanoparticles, a similar cascade to that of chitosan-treated *C. parvum* oocysts was seen, wherein the authors demonstrated that recorded changes altered the oocysts’ size, shape, interior content, and degree of staining (Ahmed et al., 2019). Additional research on other parasites confirmed the same fact. *A. annua* seeds and leaves have been observed to induce cell death in *Leishmania donovani* promastigotes (Islamuddin et al., 2014; Antwi et al., 2019). Infusions of *A. afra* at low concentrations had strong inhibitory effects on all *Plasmodium* species and stages examined (Gruessner and Weathers, 2021; Ashraf et al., 2022). *Artemisinin* was also observed to induce ultrastructural activity and damage the mitochondria of *Toxoplasma gondii* (Rosenberg et al., 2019).

In the current study, the exhibited ArPh activity against *C. parvum* could be linked to the previously reported flavonoids and phenolic acids in *A. judaica* (Goda et al., 2021). Quercetin and apigenin displayed an antiparasitic effect against *Enterocytozoon intestinalis* while naringenin and genistein were effective against *C. pavum* (Mead and McNair, 2006). Different phenolic components of *A. judaica* displayed substantial antileishmanial action via distinct pathways against diverse strains of *Leishmania*. Quercetin, fisitin, and luteolin inhibited darginase, a key enzyme in *Leishmania* (Manjolin et al., 2013). On the other hand, caffeic acid promoted amastigote apoptosis by TNF-α/ROS/NO production and reduced iron availability (Bortoleti et al., 2019). Apigenin was leishmanicidal against *L. amazonensis*-infected macrophages via the generation of ROS (Fonseca-Silva et al., 2016). It was demonstrated that *p*-hydroxybenzoic acid had an antiparasitic impact on *Entamoeba histolytica* trophozoites (Degerli and Tepe, 2015). The exact mechanism adopted by the ArPh in the damage of *C. parvum* remains of utmost interest.

The comet assay analyzed several types of DNA damage and repair in individual cells, including invertebrate and plant cells (Olive, 1999). This approach has the advantages of requiring a small number of cells, being highly sensitive to the detection of nuclear DNA damage and applying it to all eukaryotic cell types (Olive, 1999). Consequently, it was utilized to examine the apoptotic mechanism in ArPh-TO. In the present study, the comet tail moment indicated nuclear DNA of *C. parvum* produced by ArPh exposure increased in comparison to control. The tail length reflected the distance the DNA traveled from the oocyst cell and moved to its most distant location (Figure 6). This finding strongly implies that as the ArPh dose is increased, nuclear DNA fragmentation of oocysts increases.

The nuclear DNA fragmentation of ArPh-TO might be related to both parasite-specific and ArPh-specific mechanisms. While *C. parvum* lacks genes for typical molecular therapeutic targets seen in other protozoan parasites, it has numerous genes encoding unique plant-like and bacterial-like enzymes that catalyze potentially critical biosynthetic and metabolic pathways (Abrahamsen et al., 2004). *C. parvum* lacks both the tricarboxylic acid cycle and the oxidative phosphorylation steps necessary for generating metabolic energy (ATP). As a result, the parasite solely depends on anaerobic respiration (the glycolytic pathway) to generate ATP, which is essential for the parasite’s survival and the pathogenesis it causes in the host (Abrahamsen et al., 2004). Previous research has demonstrated the significance of glycolytic enzymes (pyruvate kinase) as possible therapeutic targets for the treatment of cryptosporidiosis (Eltahan et al., 2018, 2019; Khan et al., 2022). An earlier study has reported that luteolin possessed anti-leishmanial activity exerted by the induction of apoptosis through the activation of topoisomerase II-mediated kinetoplast DNA cleavage (Mittra et al., 2000). Bioflavonoids induced apoptosis and DNA damage to *Leishmania donovani* amastigotes and promastigotes (Mehwish et al., 2021). Ferulic acid-induced cellular disturbances in adult worms characterized by *in situ* DNA fragmentation, nucleosomal DNA laddering, and chromatin condensation (Saini et al., 2012).

Through the previous results we can infer that apoptosis is the mechanism of action of ArPh-oocysts toxicity as indicated by the following: i. Trypan blue staining, which was used as a basic protocol to indicate early and late apoptosis (Zhivotosky and Orrenius, 2001); ii. Comet assay, which demonstrated DNA fragmentation of oocysts, an important biochemical hallmark of apoptosis (Zhang and Xu, 2000); and iii. High ROS–mediated DNA fragmentation apoptotic properties of ArPh (Higuchi, 2003).

The animal model remains the gold standard for assessing the infectivity of oocysts (Robertson and Gjerde, 2007). Mice were fed ArPh-TO after 48 h to evaluate whether the *in vitro* impact of ArPh could reach infectivity *in vivo.* In the current study, when compared to mice that were fed with an untreated *C. parvum* oocyst infection, ArPh-TO did not induce infection in mice at 200 μg/mL (the minimum fatal dose) of ArPh *in vitro*. Similar reported data were obtained from *in vivo* experiments with *Cryptosporidium* sp. at 20 mg/mL, *A. spicigera* possessed significant anti-*Cryptosporidium* efficacy in mice (Shahbazi et al., 2021). The methanol extract of Asafoetida reduced *Cryptosporidium* sp. infection in experimentally infected mice and improved the histological alterations of small intestinal villi (Abdelmaksoud et al., 2020). The ethanolic extract of olive (*Olea europaea*) pomace, after oil pressing and phenol recovery, reduced *C. parvum* development in a reproducible manner. *Curcuma longa* L. extract significantly impacted *Cryptosporidium* oocyst shedding in *in vivo* research (Khater et al., 2017). In contrast, neither water nor ethanol extracts of propolis could eliminate the infection, but they did lower oocysts shedding (Soufy et al., 2017). The promising antioxidant activity of phenolic fraction of *A. judaica* demonstrated in the current investigation may be the responsible for the negative results of the infectivity assay, since the *C. parvum* infection is ROS-dependent (Bhagat et al., 2017). However, the role of plant extracts and phytochemicals against *Cryptosporidium* oocysts is still debatable (Almoradie et al., 2018; Ullah et al., 2020). Further clinical trials would also be encouraging in future studies.

## 5. Conclusion

This study demonstrates that the phenolic fraction of *A. judaica* L. can be a potentially medication effective against *C. parvum* oocysts by activating nuclear DNA fragmentation and destroying its morphological characteristics and the number of viable oocysts.

The benefits of using ArPh are its simple application method and its high level of safety as a herb. *A. judaica* phenolic part can be utilized to treat water systems and evident *Cryptosporidium* sp. contamination and thereby avert multiple waterborne epidemics. It can also be added to food sources to avert foodborne cryptosporidiosis or formulated into a medicine to treat humans and animals, and particularly in farm cows, to prevent cryptosporidiosis-related industrial losses.

## Data availability statement

The original contributions presented in the study are included in the article/Supplementary material, further inquiries can be directed to the corresponding authors.

## Ethics statement

The animal study was reviewed and approved by Suez Canal University's Research and Ethics Review Committee (Approval number: 5603, October 2022).

## Author contributions

SA and PK contributed to conception and design of the study. SA, EE, SE, MN, and AM organized the database. SA, EE, SE, and MN performed the statistical analysis. SA wrote the first draft of the manuscript. EE, SE, and MN wrote sections of the manuscript. SA, EE, MN, and AM funded resources of the experiment. SE funding of publication. All authors contributed to manuscript revision, read, and approved the submitted version.

## Funding

This research work was funded by Institutional Fund Projects under grant no. (IFPIP:1929-166-1443). The authors gratefully acknowledge technical and financial support provided by the Ministry of Education and King Abdulaziz University, DSR, Jeddah, Saudi Arabia.

## Conflict of interest

The authors declare that the research was conducted in the absence of any commercial or financial relationships that could be construed as a potential conflict of interest.

## Publisher’s note

All claims expressed in this article are solely those of the authors and do not necessarily represent those of their affiliated organizations, or those of the publisher, the editors and the reviewers. Any product that may be evaluated in this article, or claim that may be made by its manufacturer, is not guaranteed or endorsed by the publisher.
